# Anti-Inflammatory and Antioxidative Potential of *Aloe vera* on the Cartap and Malathion Mediated Toxicity in Wistar Rats

**DOI:** 10.3390/ijerph17145177

**Published:** 2020-07-17

**Authors:** Vivek Kumar Gupta, Abhishek Kumar, Maria de Lourdes Pereira, Nikhat Jamal Siddiqi, Bechan Sharma

**Affiliations:** 1 Department of Biochemistry, University of Allahabad, Prayagraj, U.P. 211002, India; vkgupta@allduniv.ac.in (V.K.G.); abhishek4kumar@gmail.com (A.K.); 2 CICECO-Aveiro Institute of Materials & Department of Medical Sciences, University of Aveiro, 3810-193 Aveiro, Portugal; mlourdespereira@ua.pt; 3 FCSM-Department of Biochemistry, King Saud University, Riyadh 11495, Saudi Arabia; niksiddiqi@gmail.com

**Keywords:** pesticide, hepatotoxicity, oxidative stress, antioxidants, anti-inflammatory, hepatoprotective

## Abstract

*Aloe vera* has been the most useful medicinal herb in the world since ancient times due to its vast biological effects. The presence of high content of bioactive compounds make *Aloe vera* a promising complementary and alternative agent in disease prevention. The effectiveness of *A. vera*-based medicines against pesticide toxicity has never been evaluated. It was therefore envisaged to develop an *A. vera*-based strategy to protect the non-target animals from adverse effects of the pesticides. This article illustrates the ameliorating effect of aqueous extract (AE) of *A. vera* leaves against the cartap and malathion toxicity. To evaluate the protective impact of *A. vera* against cartap (Ctp), malathion (Mtn) and a mixture of both pesticides, the animals were divided in eight groups, each containing six rats: Group 1- C (control), Group 2- AE + C, Group 3- Ctp, Group 4- Mtn, Group 5- Ctp + Mtn, Group 6- AE + Ctp, Group 7- AE + Mtn, Group 8- AE + Ctp + Mtn. Wistar rats exposed to Ctp, Mtn and Ctp + Mtn, displayed significant change in body weight. It was observed that the WBC level increased significantly in Mtn and Ctp + Mtn challenged groups. The contents of TNF-α and IL-6 in serum increased expressively in the Ctp, Mtn and Ctp + Mtn challenged groups. Rats treated with Ctp, Mtn and Ctp + Mtn displayed significant alterations in the levels of antioxidative indices (MDA, GSH, GST, GPx, SOD and CAT). Significant alterations were recorded in the activities of AST, ALT, ACP and ALP in Ctp, Mtn and Ctp + Mtn challenged groups. The histopathological results of liver supported the biochemical data. The pre-treatment of rats with the aqueous extract of *A. vera* leaves significantly protected them from the toxicity of pesticides. These results suggested that *A. vera* extract may be used as a promising natural agent for the management of pesticide induced toxicity.

## 1. Introduction

Pesticides are chemicals generally used in farming and health practices for pest control. Traces of carbamates (Cs) and organophosphates (OPs) have been observed in water, soil, grains, vegetables, and many other food products. These pesticides have also induced toxicity to animals, manifesting into numerous adverse effects in the non-target living systems. The continuous rise in the application of pesticides has always been of environmental concern. According to the recent reports, only a very small fraction i.e., 2–5% of the total pesticides applied is used to kill pests and the remaining amount is accumulated in the abiotic and biotic environmental components [1].

Currently, the Cs and OPs are being used indiscriminately for pest control. These pesticides, belonging to different groups, may induce their adverse effects in different animal systems [2]. They have a similar mode of action i.e., a negative impact on the neurotransmission system via inhibiting the acetylcholinesterase (AChE) activity [3].They disrupt the sodium/potassium balance of the nerve fibers, following the forced conduction of the nerve impulse, which leads to the development of several disease symptoms. The toxicity of OPs is manifested through the covalent binding between the phosphate (P) group OPs and the hydroxyl (-OH) group of an amino acid residue i.e., the serine present in the catalytic pocket of AChE. However, Cs interacted non-covalently with the same residues. Therefore, OPs are irreversible inhibitors [4], while Cs are reversible inhibitors [3] of AChE. In spite of this, the above-mentioned pesticides have also been described to induce oxidative stress and perturbations in the energy metabolism of mammalian systems [1].

Cs have been found to exert their adverse influence by modulating the receptors of melatonin [4] and non-covalently inhibiting the activity of AChE [3] in mammalian systems. Some reports suggested that Cs adversely influenced the xenobiotic metabolism in the hepatocytes [5]. Cartap is a member of the thiocarbamate (TC) group of pesticides, which is a subclass of the Cs. It is an analogue of nereis toxin (a neurotoxic compound extracted from *Lumbriconereis heteropoda* [6].

Malathion, a member of OPs group of pesticides, is applied to eradicate ectoparasites, rodents, insects, conserve storing food grain and eliminate the disease-causing pathogens [7]. The pesticides belonging to this group can covalently modify and inactivate the activities of esterases [8], resulting in increased activity of cholinergic system [9]. Since OPs are extremely lipophilic in nature and get rapidly assimilated in the intestine, they can reach to all body tissues, leading to generation of several pathological aberrations instantly [10]. However, an extensive literature survey suggests that there is only meager information available concerning the impact of these pesticides on the status of the inflammatory and redox systems in the mammals.

Chemicals that increase the effect of other chemicals are called as synergists. The pesticides when used in combination have been found to induce a synergistic effect while competing for a common target site [11]. The exposure of several pesticides simultaneously to different components of the environment, involving living and non-living systems, is continued [12]. Therefore, the assessment of the synergistic effects of the pesticides has always been a challenge for evaluation of the environmental health [13]. Since the anticholinesterase agents have a common mechanism of action, the additive toxicity of pesticide mixtures can be assessed by calculating the addition of the toxic potential of each chemical. In this context, some workers have reported the enhanced cumulative impact of pesticides’ mixtures in different dose combinations [14].

There has been growing attention to monitor the levels of biomarkers of pesticide toxicity in mammalian systems for detecting the adverse clinical impact of pesticides. These biomarkers are the assessable biochemical, physiological and the behavioral indices of animals, which indicate alteration, if any, in the levels of various biomolecules including the activities of certain enzymes such as esterases, β-glucuronidase, delta-aminolaevulinic acid dehydratase, glutathione-S-transferase, glutathione peroxidase, superoxide dismutase, glutathione reductase, catalase, phosphatases, transferases and many others. The cytogenetic biomarkers include aberrations in chromosomal, micronuclei, and exchange of sister chromatid whereas hematological indices include the levels of several blood contents including reduced glutathione, and malonaldehyde.

The herbal medications have been used as viable supplements with many drugs against numerous diseases, such as neurotoxicity [15], hepatic-toxicity [16], and cardiotoxicity [17]. Plants are the richest source of antioxidants [18]. *A. vera* collected in several geographic locations contained various secondary metabolites and several natural antioxidative agents [19]. However, no evidence is available to ascertain the ameliorative effects of the whole leaf of *A. vera* or any isolated compound from it in alleviation of pesticide toxicity. *A. vera* is commonly used in folk remedies as an antidiabetic, anti-oxidative, anti-inflammatory, and antimicrobial agent [20]. Ghosh et al. (2011) [21] have indicated many other significant biological functions of *A. vera*, including its antiatherosclerosis, anticarcinogenic and hypolipidemic potential. A thorough survey in the published literature reflected that the Cs and OPs pesticides exhibited the ability to generate oxidative stress [22]. However, the ameliorating impact of aqueous extract of *A. vera* leaves has never been studied against pesticides toxicity when used as an individual and/or in combinations. The present work was, therefore, carried out in order to evaluate the impact of sublethal doses of cartap and malathion in the individual and in the combination and also to monitor the ameliorating effect, if any, of the aqueous extract of *A. vera* leaves in the rats.

## 2. Materials and Methods

### 2.1. Chemicals

Malathion was secured from Hindustan Insecticides LTD. (a Government of India Enterprise located in Bathinda in Punjab, India) New Delhi-India in liquid form, having 50% W/W purity. Cartap hydrochloride was obtained from the local supplier (Dhanuka Agritech LTD., Gurgaon in Hariyana, India in association with Sumitomo Chemical Company LTD., Tokyo, Japan), as semisolid form with 50% W/W purity. Acetylthiocholine iodide (ATI) and H_2_O_2_ was purchased from TCI, Japan. Triton X-100, ethylenediaminetetraacetic acid (EDTA), pyrogallol, sodium acetate, sodium bicarbonate, sodium carbonate, sodium dihydrogen phosphate, sodium hydrogen phosphate, and NaOH were purchased from MERK. Folin-Ciocalteu reagent (FCR), 1-chloro-2,4-dinitrobenzene (CDNB), glycerol, NADH, reduced glutathione, sodium chloride, sodium pyruvate and bovine serum albumin (BSA) were obtained from SRL Pvt. Ltd., India. All other compounds used were from analytical grade.

### 2.2. Animals

The 8-week-old male Wistar rats, with an average weight of 130 ± 10 g, were bought from the CSIR-CDRI-Lucknow, India. The animals were adapted under the standard laboratory conditions. Each group of animals contained six rats in a polypropylene cage. The free access to water and food for the animals was allowed. The procedure for the experiment was strictly designed and performed following the guidelines of the Institutional Animal Ethical Committee (IAEC) of the University of Allahabad (registration number: 839/GO/Re/04/CPCSEA).

### 2.3. Collection of Plant and Storage of Extract

Fresh leaves of *A. vera* (L.) from the *Asphodelaceae* family were collected locally in the Botanical Garden of the University of Allahabad-Prayagraj, India, during April 2018 and washed thoroughly to free it from any other organic or non-organic matter. Plants were identified and confirmed by Department of Botany, Allahabad University, India. The plant material was dried out in the shade at room temperature. Dried leaves were powderized, followed by the preparation of the extract (1:10 W/V; ratio of *A. vera* powder and water). The aqueous extract of leaves of *A. vera* was lyophilized to a dry powdered material and stored at −20 °C.

### 2.4. Treatment of Animals and Animal Care

The animal treatment was attempted with a subacute dose of pesticides (cartap, malathion and their mixture). The ameliorative impact of *A. vera* leaf (aqueous extract) i.e., AE was also endeavored. Animals were separated into 8 groups. Group 1 was served as control (C), had unrestricted access to feed and water only; Group 2 was the control (AE + C), treated with plant extract (*A. vera*), received 420 mg kg^−1^ body weight; Group 3 was challenged with cartap (Ctp), received 10% LD_50_ (29 mg kg^−1^ body weight) of cartap; Group 4 was challenged with malathion (Mtn), received 10% LD_50_ (29 mg kg^−1^ body weight) of malathion; Group 5 was challenged with the combination of cartap and malathion (Ctp + Mtn), received a mixture of 5% LD_50_ (14.5 mg kg^−1^ body weight) of each pesticide; Group 6 contained animals treated with the plant extract (*A. vera*) i.e., 420 mg kg^−1^ body weight followed by cartap (10% LD_50,_ i.e., 29 mg kg^−1^ body weight) (AE + Ctp), Group 7 included treatment with the plant extract (*A. vera*) (420 mg kg^−1^ body weight) followed by malathion (10% LD_50,_ i.e., 29 mg kg^−1^ body weight) (AE + Mtn). Group 8 received 420 mg kg^−1^ body weight of *A. vera* extract followed by a mixture of both pesticides (5% LD_50_;14.5 mg kg^−1^ body weight of each) (AE + Ctp + Mtn).

The oral gavage for all groups was performed for up to 15 days, with a regular interval of 24 h. During treatment, the percentage change in body weight and clinical symptoms were also noted. At the end, all animals were euthanized following the guidelines of Institutional Animal Ethical Committee.

### 2.5. Assessment of Rat Blood on Hematological Indices and Preparation of Blood Sample

After sacrificing the animals, blood from the rat’s heart was collected in sterile heparinized vials. The rat blood was investigated for complete blood count (CBC). The blood was also processed for erythrocytes, and serum isolation in non-heparinized tubes. The serum was stored at −20 °C for further use for biochemical investigations.

### 2.6. Estimation of Inflammatory Markers (TNF-α and IL-6)

The estimation of TNF-α in addition to the levels of IL-6 in the blood serum of rats exposed to cartap, malathion and their mixture was achieved by the GENLISA^TM^ ELISA Kits procured from KRISHGEN Biosystems, Worli, Mumbai, India. The procedures were followed as mentioned in the kit protocol.

### 2.7. Estimation of Malondialdehyde (MDA)

The level of lipid peroxidation (LPO) was assessed by the method of Niehaus and Samuelsson (1968) [23]. The result was presented as nmol MDA/mg protein considering the ε (extinction coefficient) at 1.56 × 10^5^ M^−1^ cm^−1^.

### 2.8. Estimation of Glutathione (GSH)

The GSH content was determined in serum by Ellman et al. 1959 method [24], modified by Sedlak and Lindsay (1968) [25]. The optical density (OD) of the colored complex derived from the supernatant of reaction mixture was supervised at 412 nm. The result was expressed in µg of GSH/mg of protein.

### 2.9. Activity Assay of Glutathione-S-Transferase (GST)

The estimation of GST activity was conducted following Habig et al. (1974) [26] method and change in OD was recorded at 340 nm in the interval of 30 s for 3 min. The enzyme activity was calculated by ε (9.6 × 10^3^ µmol^−1^ cm^−1^). The results for the specific enzyme activity were expressed in µmoL/mL/min/mg protein.

### 2.10. Activity Assay of Glutathione Peroxidase (GPx)

The GPx activity in RBCs hemolysate was assayed by the method of Pagalia and Valentine (1967) [27]. The rate of oxidized GSH formation was monitored spectrophotometrically using UV-visible double-beam spectrophotometer (i.e., ThermoScientific Spectroscan UV2700) at 340 nm with NADPH. The unit for enzyme activity was denoted as IU/mg Hb.

### 2.11. Activity Assay of Superoxide Dismutase (SOD)

The activity assay for the estimation of SOD was conducted by the method of Marklund and Marklund (1974) [28]. The OD of pyrogallol auto-oxidation was spectrophotometrically monitored at the interval of 30 s for 3 min at 412 nm. The unit of enzyme activity was expressed as inhibition of pyrogallol auto-oxidation at 50% min^−1^.

### 2.12. Activity Assay of Catalase (CAT)

The activity assay of CAT was done according to Beers and Sizer (1952) method [29]. The decrease in OD of H_2_O_2_ consumption was observed at the intervals of 30 s for 3 min at 240 nm. The unit of activity of the enzyme was stated as H_2_O_2_ decomposed in µm min^−1^. The ε (43.6 M^−1^ cm^−1^) of H_2_O_2_ was used to calculate the enzyme activity.

### 2.13. Activity Assay for Transaminases and Phosphatases

The activity assays of transaminases, such as aspartate transaminase (AST) and alanine transaminase (ALT), were performed using the method described by the Reitman and Frankel (1957) [30]. The OD of total volume of the reaction mixture (TRMV; 3 mL) was observed at 510 nm. The TRMV without enzyme was considered as control.

The activities of ACP and ALP were measured by the method of Goldberg and Barka (1962) [31]. The variation in the OD of TRMV was monitored at 405 nm for 3 min at intervals of 30 s. The unit of specific activity of the enzyme was denoted as µmol/mL/min/mg protein.

### 2.14. Assessment of Total Protein Content (TPC)

The TPC in different samples was estimated according to the Lowry et al. (1951) [32] method using BSA as standard protein.

### 2.15. Histological Analysis of Liver

The liver tissues were quickly removed from the experimental animals, followed by washing with buffered saline, blotted dry, and weighed. The fixation of tissues in Bouin’s fixative was performed overnight, followed by continuous washing up to 24 h in running tap water. This process was trailed by dehydration of tissues in the graded ethanol (i.e., from 30–100%, *v*/*v*). The dehydrated tissues were fixed in paraffin wax. The embedded tissues were serially sectioned (10 μm) with the SPINCON rotary microtome (model number SP-1120/SP-1120A). The wax ribbon containing the slices of liver tissue was distributed on albumin coated sterilized glass slides, followed by stretching with the help of hot plate. The stretched sections were used for the staining using the eosin and hematoxylin dyes, using the standard procedures. These slides with tissue sections were mounted with dibutyl phthalate polystyrene xylene and images were observed by a light microscope (model-YJ-2016). All tissue sections were scrutinized for the extent of hemorrhage, vacuolar degeneration, vascular congestion, dilation of sinusoids, and the necrosis. Each slide of liver was examined, and the aberrations were scored using the scale of none (-), severe (+++), moderate (++) and mild (+).

### 2.16. Statistical Analysis

The data was analyzed by GraphPad Prism version 5.01, San Diego, CA, USA, using one-way analysis of variance (ANOVA). The Bonferroni’s Multiple Comparison Test was preferred to compare different groups. The result was considered significant at *p* ≤ 0.05. The histological analysis of hepatic tissues was done using the Dewinter microscopic camera DGI810 (Dewinter Optical Inc., New Delhi, India) and Dewinter biowizzard imaging software (Dewinter Optical Inc., New Delhi, India) associated with the Dewinter microscope (model-YJ-2016).

## 3. Results

### 3.1. Clinical Symptoms Induced by Pesticides and Effect of A. vera Leaf Extract

After the treatment of pesticides up to 15 days, no deaths were observed in any group. However, pesticide-challenged groups displayed varying degrees of clinical symptoms after dosing. The clinical symptoms detected in the pesticide challenged animals included gathering and hugging with mild shock. When compared to control, Wistar rats challenged with cartap, malathion and their mixture displayed significant changes in body weight of 24.08%, 30.87% and 31.42%, respectively. Though, the pre-administration of *A. vera* extract in the groups challenged with pesticides registered an ameliorating impact on the alteration in their body weight (Table 1).

### 3.2. Effect of Pesticides and the Aqueous Extract of A. vera Leaf on the Status of Hematological Parameters of Rat

To assess the effect of pesticides individually and mixed at the sublethal doses, rats were administered orally for 15 days, as mentioned in Section 2. The rats challenged with pesticides were also pre-administered orally with the aqueous extract of *A. vera* leaves for the evaluation of their protective effect, if any. The results as summarized (Table 2) demonstrated that the pesticide-challenged rats did not show any significant alterations in terms of any of the hematological parameters, except for the total WBC count compared to the control. The level of total WBC count was altered significantly in malathion and a mixture of groups challenged with cartap and malathion only. The extract of *A. vera* itself ensured non-significant changes in hematological parameters among the groups (Table 2).

### 3.3. Effects of Pesticides and the A. vera Leaves on TNF-α and IL-6 Levels in Rat Blood Serum

**Figure 1:**
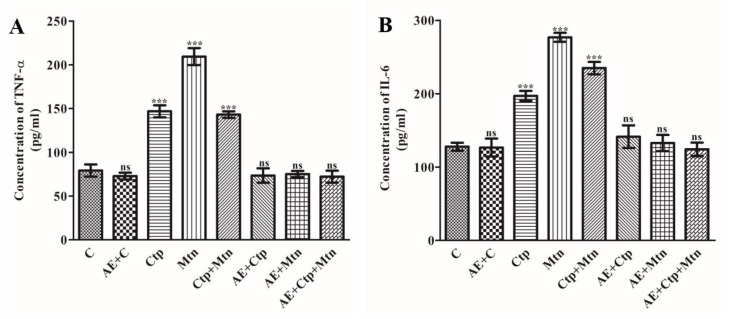
Effects of cartap, malathion and the aqueous extract of *A. vera* leaves on the levels of pro-inflammatory markers i.e., (**A**) TNF-α and (**B**) IL-6, in the rat blood serum. The procedures for the administration of pesticides, aqueous extract of *A. vera* leaves and the assay for TNF-α and IL-6 were mentioned in Materials and methods. The unit of the concentration of TNF-α and IL-6 were expressed as pg mL^−1^. The data represent mean ± SD of 3 independent experiments. The values were significant at *p* < 0.05 as compared to control group.

Cytokines are small proteins or peptides that function as immunomodulatory representatives and are also associated with paracrine, autocrine and endocrine signaling. Cytokines include tumor necrosis factors, chemokines, interleukins, lymphokines and interferons. They play a significant role in health and disease, such as inflammation, trauma, immune response, and cancer. The level of pro-inflammatory cell cytokines is considered as the potential markers of inflammation and cell damage. The concentrations of pro-inflammatory cytokines (TNF-α and IL-6) in the blood serum of rats exposed to sublethal doses of pesticides were altered significantly (Figure 1A,B). The levels of concentration of pro-inflammatory cytokines increased significantly (*p* < 0.05) in the rat serum by 85.44, 163.76, and 80.77 % for TNF-α as well as 53.86, 116.15, and 83.37 % for IL-6, respectively, in the groups challenged with cartap, malathion and their combination. When compared to the impact of each pesticide, their combination did not confirm any further rise in the pro-inflammatory markers. The levels of these pro-inflammatory markers were observed to be protected by the pre-administration of *A. vera* extract.

### 3.4. Effect of Pesticides and the Aqueous Extract of A.vera Leaves on the Contents of MDA and GSH in Blood Serum of Rat

To assess the effect of cartap, malathion and the mixture of these compounds on the levels of MDA and GSH in the rat blood, the animals were exposed as mentioned in Section 2. The pesticide challenged rats were also administered with *A. vera* extract to assess its protective effect if any. The results shown in Table 3 reflected that rats challenged with these two pesticides significantly (*p* < 0.05) rise the MDA and decrease GSH levels in rat serum when administered alone or in mixture. The *A. vera* leaf extract did not exert any noteworthy changes in the contents of MDA (3.73%) and GSH (5.30%). Each of these pesticides used in individual (cartap / malathion) or in co-exposure caused a significant enhancement in MDA (i.e., 27.48, 42.52, and 25.33%, respectively) and a decline in GSH levels (i.e., 22.44, 26.12, 23.67%, respectively). The individual challenge of malathion exerted a relatively high level of change, with an increased MDA content (42.52%) and decreased GSH content (26.12%). When rats were pre-treated with *A. vera* leaf extract, their exposure to cartap or malathion did not alter MDA and GSH contents (Table 3).

### 3.5. Effect of Pesticides and A. vera Leaves Extract on GST, GPx, SOD and CAT Activities in Rat Blood Serum

**Figure 2:**
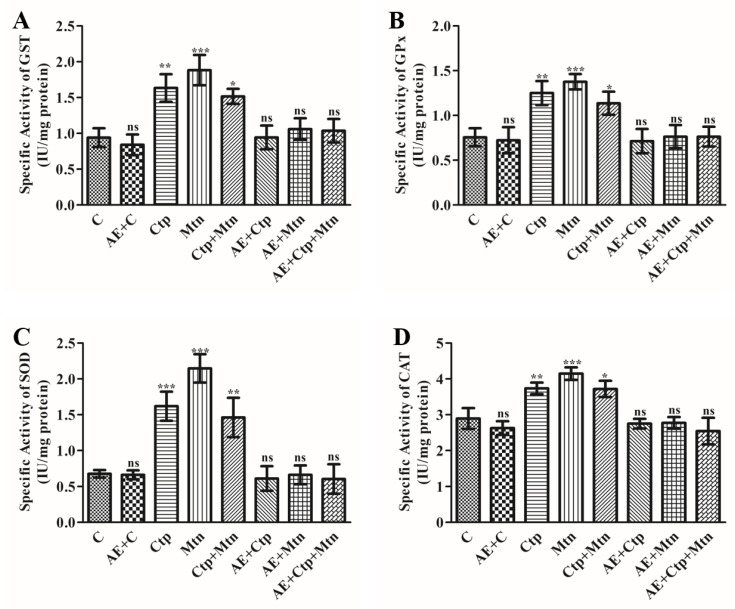
Effect of cartap, malathion and aqueous extract of *A. vera* leaves on the levels of antioxidative enzymes rat blood serum. The activity of enzymes (**A**) GST, (**B**) GPx, (**C**) SOD, and (**D**) CAT were determined in the rat blood serum as mentioned in Materials and methods. The unit of enzyme activity was expressed as IU mg^−1^ protein. The data represent mean ±SD of 3 independent experiments. The values were significant at *p* < 0.05 as compared to control group.

To evaluate the effect of pesticides on the activities of enzymes viz; GST, GPx, SOD and CAT, the assays for their activities were carried out using serum from rats exposed to cartap, malathion and a mixture of both as mentioned in Section 2. The results indicated that rats challenged with these pesticides significantly raised the functions of GST (Figure 2A), GPx (Figure 2B), SOD (Figure 2C), and CAT (Figure 2D). The percentage rise in the actions of these enzymes was computed at 73.82, 100.31 and 61.38% for GST; 65.19, 81.97, and 50.25% for GPx; 139.14, 217.13 and 115.80% for SOD, and 29.02, 43.36, and 28.54% for CAT in the corresponding pesticide-challenged groups (cartap, malathion and cartap + malathion challenged groups, respectively). When rats were pre-treated with *A. vera* leaves extract, these groups indicated complete protection against toxicity induced by pesticides. Their activities (GST, GPx, SOD, and CAT) were completely protected due to pre-treatment of the extract (Figure 2). The treatment of *A. vera* leaves extract showed almost no variation in these indices even up to 15 days.

### 3.6. Effect of Pesticides and A. vera Leaves Extract on the Activities of Transaminases and Phosphatases in the Rat Blood Serum

**Figure 3:**
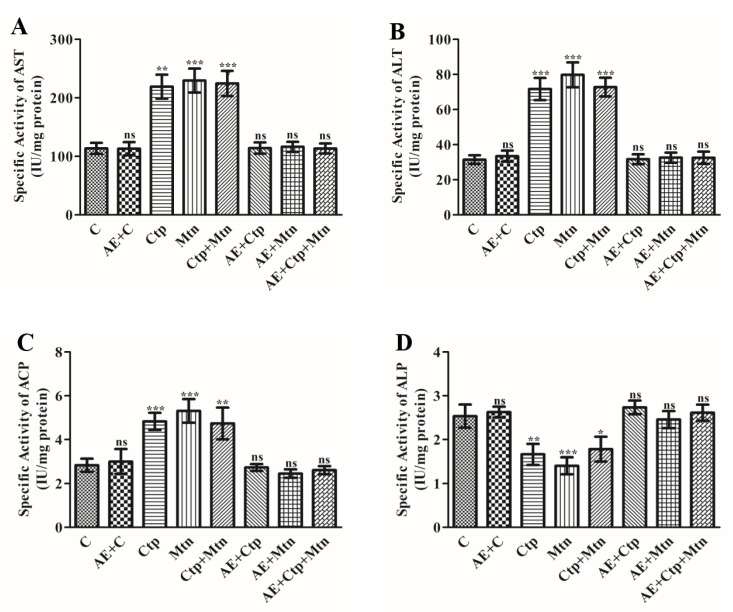
Effect of cartap and malathion as well as the aqueous extract of *A. vera* on the activity of transferases in rat blood serum. The activities of enzymes (**A**) AST, (**B**) ALT (**C**) ACP and (**D**) ALP were estimated in rat blood serum as mentioned in Materials and methods. The unit of enzyme activity was expressed as IU mg^−1^ protein. The data represent mean ± SD of 3 independent experiments. Values were significant at *p* < 0.05 as compared to control group.

Transaminases (ALT and AST) and phosphatases (ALP and ACP) are cytosolic enzymes considered as potential biomarkers of the status of hepatic function. The consequence of exposure of sublethal concentrations of pesticides on the hepatic function of rat was evaluated by estimating the activities of ALT, AST, ALP and ACP in serum (Section 2). The protective impact of *A. vera* leaf extract was also evaluated. The results indicated a substantial upsurge in activities of AST by 92.10, 101.75 and 97.36% (Figure 3A), ALT in 127.61, 153.33, 131.11% (Figure 3B), ACP in 70.07, 86.97 and 66.90% (Figure 3C) and decreased ALP by 34.64, 44.88, and 29.92% (Figure 3D) in their corresponding groups (Ctp, Mtn, and Ctp + Mtn) in comparison to the control of rat blood serum. The mixture of pesticides i.e., (Ctp + Mtn) also exhibited a marked increase in their impacts. However, the pretreatment of extract of *A. vera* leaves indicated a protective effect.

### 3.7. Effect of A. vera Leaves Extract on Pesticides Treated Rat Liver Histology

**Figure 4:**
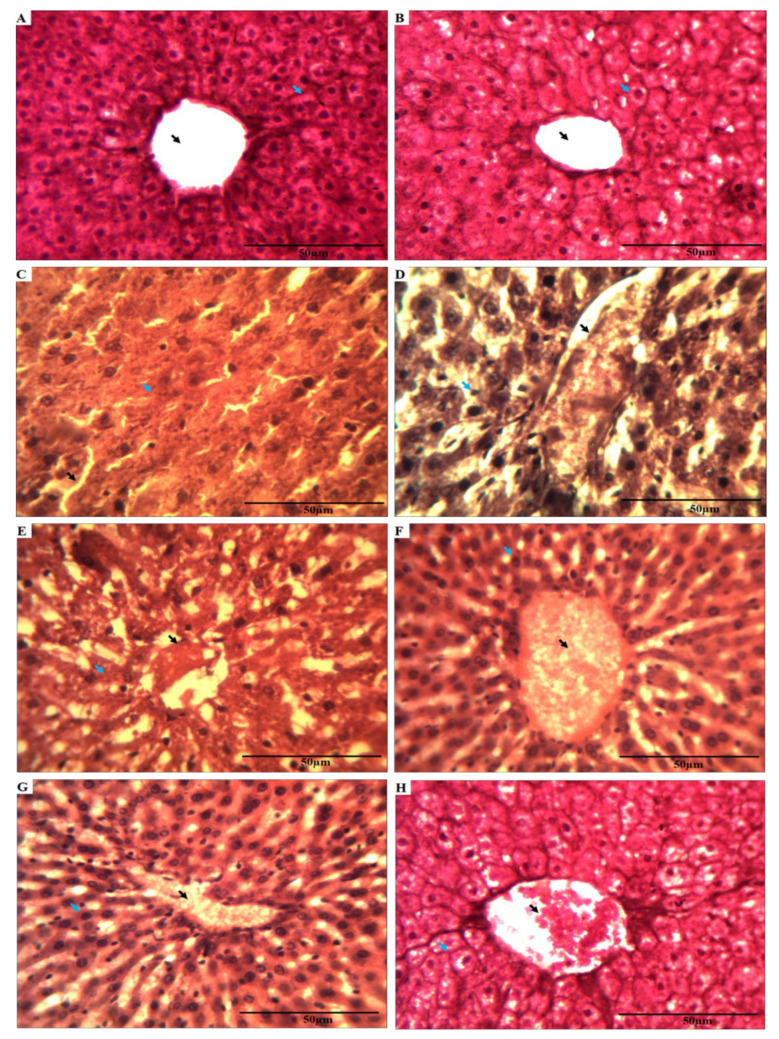
Histological images of rat liver exposed to pesticides and aqueous extract from *A. vera* leaves for 15 days. Photomicrograph of a tissue from (**A**) C group, (**B**) AE + C group, (**C**) Ctp challenged group, (**D**) Mtn challenged group, (**E**) Ctp + Mtn challenged group (**F**) AE + Ctp treated group, (**G**) AE + Mtn treated group, (**H**) AE + Ctp + Mtn treated group. The black arrow indicates changes in central vein, and the blue arrow indicates the disorganization in hepatic cells. Abbreviations: C: Control, AE + C: *Aloe vera* treated control, Ctp: cartap, Mtn: malathion, Ctp + Mtn: mixture of cartap and malathion, AE + Ctp: *Aloe vera* treated with cartap, AE + Mtn: *Aloe vera* extract treated with malathion, AE + Ctp + Mtn: *Aloe vera* extract treated with the mixture of cartap and malathion.

The liver tissues dissected from rats challenged with sublethal doses of pesticides for 15 days were processed and analyzed for histological aberrations, if any, as described in Section 2. Light microscopic observation of hepatic tissues as shown in Figure 4A,B demonstrated normal hepatic histological structure. The hexagonal lobules existed around the central vein (CV). It had portal triad of bile duct (BD), portal vein (PV) and hepatic artery (HA). The disorganization in the structure of the CV and hepatic cords appeared in liver tissues of rats exposed to cartap (Figure 4C), malathion (Figure 4D), and their combination (Figure 4E). The histological sections from rat liver pre-treated with *A. vera* leaves’ aqueous extract exhibited moderate protection (Figure 4F–H) against pesticide damage. The scores allotted to the changes in hepatic cellular organization of rat were shown in Table 4.

## 4. Discussion

The extensive application of pesticides has increased the exposure to non-target invertebrates, birds, and mammals including humans [7], by dermal, oral and by inhalation routes [33]. The use of pesticide is a double-edged weapon in agricultural practices. Pesticides could control the target pests and help increase the crop yields, whereas they would contaminate water, soil, air and foodstuffs. It caused toxicological consequences in several nontarget animals including the human’s health [34]. Exposure to pesticides in living systems has been revealed to alter the vital functions of their organs associated to the cholinergic system, energy metabolism and liver. The perturbations in these parameters may lead to the several ailments interrelated with functions of these marker enzymes. Therefore, there is an urgent necessity to protect the non-target living systems including mammals to keep them safe from occurrence of liver diseases [35] and the neurodegenerative disorders. The plant extract-based therapy has proved to be highly promising in this context [36].

Among different biological markers used for assessment of pesticide toxicity, the, body weight is considered as a significant morphological parameter. The data from the present study reflected substantial variations in the body weights of rats exposed to pesticides, which may be associated to the cholinergic deficiency and oxidative stress. Our findings supported the results presented by others [37,38]. However, the pretreatment of rats by *A. vera* leaf extract markedly attenuated changes in body weight of animals exposed to pesticides. The current effect of the plant extract can be allied with the several antioxidative moieties present in the preparation.

The pesticide induced alterations in different hematological parameters have been indicated earlier by some workers [39]. Our findings have clearly displayed that pesticide exposure reflected a non-significant change in these parameters, except for WBCs which was significantly increased in the blood of rats due to pesticides-challenge. Similar to our observations, Kalender et al. (2006) [40] have demonstrated an increase in WBCs count in rats challenged with malathion. Although the rise in WBC count in rats challenged with pesticides in this study was significant, the leucocytosis has been implicated in the strictness of stress [39]. Thus, leukocytosis may reflect the response of immune system to the stress caused by pesticides. In addition, it was revealed that OPs induce alterations in the hematological parameters [40,41]. Proinflammatory cytokines are low molecular mass proteins secreted mainly by the activation of macrophages and lymphocytes [42]. Environmental contaminants, like pesticides, can potentially initiate the production of these marker cytokines [43,44]. The dysregulated production of cytokines has been linked with many inflammatory conditions [45]. It is known that stress induced by xenobiotics generates inflammatory responses through the activation of factors sensitive to redox factors, such as NF-κB [46]. In our study, this transcription factors would have been activated due to enhanced oxidative stress induced by cartap, malathion and a mixture of both compounds. The increased levels of TNF-α and IL-6 in rats exposed to pesticides in the present investigation thus indicate the generation of inflammation by cartap, malathion or a combination of both exposures. Furthermore, the present study revealed the ability of *A. vera* to attenuate the adverse impact of cartap, malathion and the inflammation induced by both; as the pre-treatment of animals with *A. vera* leaves extract significantly protected the levels of TNF-α and IL-6 in rats exposed to pesticides.

The response against the inflammation occurs when the tissues are damaged. The injured cells release chemicals, including bradykinin, histamine, and prostaglandins causing leakage of blood fluid into tissues, thereby causing swelling. This helps to separate foreign particles from an additional association with body tissues and enhance phagocytosis. Cyclooxygenase (COX) enzymes (also known as prostaglandin-H2-synthases) act as catalysts in the production of prostaglandins (highly active pro-inflammatory mediators) from arachidonic acid during the inflammatory response. These responses may occur either by lipoxygenase pathway or by COX pathway. The glycoproteins present in *A. vera extract* have been shown to inhibit the COX-2 activity [47]. The administration of *A. vera* extract has been displayed to result in proliferative and phagocytic activity caused by the inhibition of COX pathways and reduction of prostaglandin-E2 [48,49]. Levels of transcription of albumin and TNF-genes have been shown to be linked in the early phase of the acute inflammatory response. *A. vera* was found to contain an inhibitory potential against the inflammatory process of burn injury [50]. Phytosterols (campesterol, β-sitosterol, lupeol, and cholesterol) present in *A. vera* have demonstrated as enhancer of the anti-inflammatory actions [51]. The plant also contained anthraquinones and chromones, which have anti-inflammatory potential on murine macrophages [52]. The *A. vera* extracts prepared in ethyl acetate and the ethanol have been indicated to be helpful in the therapy of corneal inflammation [53]. Furthermore, in the inflammation process, mediators derived from the endothelium and other factors stimulate the adhesion of leucocytes. It has been shown that *A. vera* extract contains a high content of flavonoids which are known to suppress this leucocyte adhesion, thereby, stopping the liberation of inflammatory mediators and oxidants [54]. Due to the high content of flavonoids, *A. vera* leaves may have the potential to suppress leucocyte adhesion and therefore, play an additional role in its anti-inflammatory functions as shown by the current study. In addition, the ingested extract of *A. vera* leaves may undergo chemical modifications and the resulting products together with the native phytochemicals might contribute significantly to their protective biological effects.

The exposure of animals to any xenobiotics results in the generation of excess free radicals (ROS), which results in a marked decline in the cellular antioxidants, thereby inducing disordered redox system. The LPO is known to produce many TBARS; the marker of oxidative damage including MDA [55]. The results generated from this study illustrated significantly enhanced MDA level in the rat serum when the animals were subjected to the pesticides exposure. These findings were corroborated with those from several other workers [10,56].

However, this study indicated that when animals were pre-treated with the *A. vera* extract, a significant improvement was observed on rats against pesticide induced toxicity, as the level of MDA came close to control. The extract of *A. vera* leaves have been shown to attenuate the toxicity of pesticides in rat serum due to their strong antioxidative flair [57].

The GSH (a tripeptide) is a potential non-enzymatic cellular antioxidant known as a key biomarker of stress in the living systems [58]. Our results, showing a significant decrease in the GSH content, as recorded in rat serum, suggested the development of oxidative stress in animals treated with the pesticides. This finding is in agreement with the data reported by Selmi et al. (2018) [10]. The pre-administration of *A. vera* leaves extract caused a significant restoration of the GSH content in the blood serum of rats treated with pesticides.

Enzymes of the antioxidative system are actively involved in maintaining the oxidant/antioxidant homeostasis and in the detoxification of xenobiotics in the biological system. The glutathione-S-transferases (GSTs) catalyze the reaction involving conjugation of reduced-glutathione to any xenobiotics leading to the detoxification [59] and GPx actively tangled in the oxidation reduction reaction of GSH. The SOD, responsible for catalyzing the biochemical reaction involving the dismutation of the superoxide radicals in H_2_O_2_, which further degraded into H_2_O and O_2_ molecules by CAT [60]. A network of these enzymes is involved in the instant removal of free radicals formed due to xenobiotic stress. Any perturbations in their activities caused by pesticides may result into the emergence of serious consequences at the pathophysiological levels in mammals [16,17]. Our results indicated notable variations in the activities of these enzymes due to exposure to pesticides. The altered activities of these enzymes indicate the failure of the antioxidative defense system. Our findings were supported by similar observations reported when rats were treated with malathion and methicarb [10]. However, the pre-treatment of *A. vera* extract for rats reflected a significant level of protection against the activities of these enzymes.

The functions of phosphatases and transaminases have been potential biomarkers to assess tissue damage [61]. Whenever hepatocytes are damaged, several enzymes located inside, including AST, ALT, ACP, and ALP are secreted into the blood [62]. The sharp increase in the activities of transaminases and phosphatases in the blood of rats treated with insecticide indicated hepatic damage in the present study. The toxicity of OPs has been verified by other workers who have shown rise in the activities of AST, ALT, ACP, and ALP [39]. The reports from other authors also indicated similar observations [38,63]. The transaminases are recognized to play vital roles in regulating physiological processes, catalyzing trans-amination reactions to facilitate the metabolism of xenobiotics and other macromolecules [64]. The data from our study revealed that pretreatment with *A. vera* leaf’s extract could significantly protect these marker enzymes from the adverse effects of pesticides. Al-Shinnawy et al. (2014) [63] have also shown similar findings using *A. vera* juice in young male rabbits. The *A. vera* extract was found to contain hepatoprotective impact against cartap and malathion mediated toxicity in the experimental rats [38].

The rats exposed to pesticides resulted in substantial injury to the liver tissues, as noticed in the present investigation. Such tissue damage may arise owing to the adverse influence of both pesticides. These findings were verified by the observations recorded by other researchers [38,65]. Handy et al. (2002) [66] have reported that the rats exposed to malathion exhibited injuries to hepatocytes. By conducting histological examination, these workers have displayed that the carbamate pesticide (cartap) and organophosphate (malathion) may cause degeneration of hepatocytes. In the present study, the protection against pesticide intoxication was offered by the aqueous extract of *A. vera* leaves has been demonstrated by other workers [10,38,67].

## 5. Conclusions

Pesticides are known to cause serious ailments in the not-target animals, including mammals. Liver plays a significant role in the metabolism of xenobiotics, and hence it is responsible for their detoxification. Liver, therefore, serves as the main target for actions of various anthropogenic chemicals, including pesticides. The results from present study have revealed that the sub-lethal dosage of cartap and malathion, when used individually or in their combination, caused serious perturbations in the levels of hematological, pro-inflammatory (i.e., TNF-α and IL-6), and oxidative markers (cellular antioxidants i.e., MDA, GSH, GST, GPx, SOD and CAT), as well as in the activities of transaminases (AST and ALT), and phosphatases (ACP and ALP) in the rat blood serum. The histological investigation of rat liver treated with pesticides registered severe damage to hepatocytes. Interestingly, the administration of aqueous extract of *A. vera* leaves displayed remarkable level of protection of rat tissues against the pesticides mediated damage, which could be allied to antioxidants present in *A. vera.* These findings suggested that the natural ingredients isolated from *A. vera* leaves might be used as cost effective therapeutic supplements to significantly reduce the xenobiotics-induced burden of oxidative stress and cytotoxicity. The information generated from this investigation may also be useful in the development of the safe and effective formulation of pesticides, in the best interest of environmental health.

## Figures and Tables

**Table 1:** Impact of cartap, malathion and *A. vera* extract on rat body weight, levels of MDA and GSH.

P (g)	C	AE + C	Ctp	Mtn	Ctp + Mtn	AE + Ctp	AE + Mtn	AE + Ctp + Mtn
**BWI**	132.20 ± 5.31	135.20 ± 3.38	131.80 ± 1.06	132.30 ± 1.86	135.7 ± 2.99	139.30 ± 2.06	130.20 ± 2.03	136.80 ± 2.46
**BWF**	141.30 ± 4.74	141.60 ± 1.37	195.30 ± 3.83	214.00 ± 4.88	221.0 ± 2.86	144.78 ± 3.40	151.05 ± 2.79	145.18 ± 3.18
**BW#**	+3.44%	+2.36%	+24.08%	+30.87%	+31.42%	+1.96%	+8.00%	+3.06%

Values are presented as mean ± SD of 6 rats. Abbreviations: P (g): parameters in grams; C: control; AE + C: *A. vera* treated control; Ctp: cartap treated; Mtn: malathion treated; Ctp + Mtn: cartap + malathion treated; AE + Ctp: cartap with pretreatment of *A. vera*; AE + Mtn: malathion with pretreatment of *A. vera*; AE + Ctp + Mtn: cartap + malathion with pretreatment of *A. vera*.

**Table 2:** Effect of cartap, malathion and *A. vera* extract on hematological parameters of the Wistar rat in different treatment groups.

HP	C	AE + C	Ctp	Mtn	Ctp + Mtn	AE + Ctp	AE + Mtn	AE + Ctp + Mtn
**Total WBC Count (** **×10^3^/mm^3^)**	5.233 ± 1.002	3.933 ± 1.155 ns	7.767 ± 0.585 ns	12.67 ± 1.021 ***	11.33 ± 1.159 ***	5.63 ± 1.514 ns	4.4 ± 0.866 ns	6.8 ± 0.964 ns
**LYM (%)**	54.93 ± 7.242	38.43 ± 20.6 ns	75.77 ± 5.828 ns	68.0 ± 9.001 ns	59.4 ± 22.69 ns	75.1 ± 8.163 ns	44.13 ± 6.067 ns	63.8 ± 7.9 ns
**MON (%)**	15.13 ± 2.743 ns	15.8 ± 1.825 ns	13.0 ± 4.244 ns	12.83 ± 1.914 ns	11.97 ± 5.514 ns	11.10 ± 3.305 ns	17.77 ± 2.715 ns	13.17 ± 0.923 ns
**GRAN (%)**	29.93 ± 4.508 ns	45.8 ± 18.83 ns	11.23 ± 1.589 ns	19.17 ± 7.821 ns	28.63 ± 17.18 ns	13.8 ± 4.859 ns	38.1 ± 8.73 ns	23.03 ± 8.822 ns
**MCH (mmg)**	19.63 ± 0.9292	17.53 ± 0.7767 ns	17.73 ± 2.454 ns	19.23 ± 2.914 ns	19.33 ± 2.369 ns	19.43 ± 0.7234 ns	19.93 ± 0.3786 ns	21.5 ± 0.8718 ns
**MCHC (mg/dL)**	40.5 ± 0.2646	41.13 ± 0.2062 ns	37.47 ± 7.508 ns	38.23 ± 6.525 ns	38.23 ± 5.659 ns	38.6 ± 2.858 ns	41.63 ± 1.137 ns	40.1 ± 0.2 ns
**HGB (g/dL)**	12.8 ± 0.1 ns	12.33 ± 0.4933 ns	13.73 ± 0.8083 ns	10.03 ± 3.754 ns	10.9 ± 2.707 ns	11.83 ± 0.6807 ns	13.5 ± 0.1 ns	11.67 ± 1.242 ns
**MCV (µm^3^)**	48.47±1.935	42.6 ± 1.587 ns	47.77 ± 3.493 ns	50.47 ± 1.405 ns	50.63 ± 1.358 ns	50.37 ± 2.023 ns	47.9 ± 2.17 ns	53.53 ± 2.369 ns
**HCT (%)**	31.6 ± 0.2	29.97 ± 1.097 ns	36.87 ± 5.054 ns	25.83 ± 6.643 ns	27.8 ± 2.787 ns	30.87 ± 4.2 ns	32.43 ± 0.8083 ns	29.07 ± 2.974 ns
**Total RBC Count (** **×10^6^/mm^3^)**	6.527 ± 0.2608 ns	7.033 ± 0.1106 ns	7.697 ± 0.4801 ns	5.12 ± 1.309 ns	5.5 ± 0.6872 ns	6.11 ± 0.5747 ns	6.78 ± 0.14 ns	5.453 ± 0.8198 ns
**PLT** **(×10^3^/mm^3^)**	399.7 ± 72.61	463.7 ± 54.17 ns	584.7 ± 11.37 ns	387.7 ± 119.3 ns	339.7 ± 207.6 ns	311.3 ± 115.8 ns	248 ± 54.62 ns	252.3 ± 40.5 ns
**MPV (µm^3^)**	5.967 ± 0.5508	5.467 ± 0.05773 ns	5.367 ± 0.1155 ns	5.5 ± 0.3 ns	5.567 ± 0.05773 ns	6.133 ± 0.5686 ns	7.3 ± 0.8185 ns	6.133 ± 0.6506 ns
**PCT (%)**	0.2377 ± 0.03968 ns	0.253 ± 0.02696 ns	0.314 ± 0.001732 ns	0.2743 ± 0.04676 ns	0.1503 ± 0.1032 ns	0.195 ± 0.08479 ns	0.1973 ± 0.05173 ns	0.1697 ± 0.02566 ns

Abbreviations: HP: hematological parameters; C: control; AE + C: *A. vera* treated control; Ctp: cartap treated; Mtn: malathion treated; Ctp + Mtn: cartap + malathion treated; AE + Ctp: cartap with pretreatment of *A. vera*; AE + Mtn: malathion with pretreatment of *A. vera*; AE + Ctp + Mtn: cartap + malathion with pretreatment of *A. vera*; WBC: white blood cells; LYM %:percent lymphocyte; MON %:percent monocytes; GRAN %: percent granulocyte; RBC: red blood cells; HGB: hemoglobin; HCT: hematocrit; MCV: mean corpuscular volume; MCH: mean cell hemoglobin; MCHC: mean corpuscular hemoglobin concentration; PLT: platelets; MPV: mean platelet volume; PCT: platelet crit; ns: nonsignificant; * *p* < 0.05; **p < 0.005; *** *p* < 0.005.

**Table 3:** Effect of cartap, malathion and *A. vera* extract on MDA and GSH in the Wistar rat blood serum.

P	C	AE + C	Ctp	Mtn	Ctp + Mtn	AE + Ctp	AE + Mtn	AE + Ctp + Mtn
**MDA**	30.24 ± 2.00	31.37 ± 2.24 ns (+3.73%)	38.55 ± 2.79 ** (+27.48%)	43.10 ± 1.65 *** (+42.52%)	37.90 ± 1.95 ** (+25.33%)	31.64 ± 2.49 ns (+4.62%)	30.23 ± 1.49 ns (−0.03%)	33.46 ± 1.50 ns (+10.64%)
**GSH**	245.0 ± 17.7	258.0 ± 19.80 ns (+5.30%)	190.0 ± 20.80 * (−22.44%)	181.0 ± 15.10 * (−26.12%)	187.00 ± 10.70 * (−23.67%)	224.00 ± 18.10 ns (−8.57%)	215.00 ± 17.80 ns (−12.24%)	231.0 ± 17.60 ns (−5.71%)

Abbreviations: P: parameters; C: control; AE + C: *A. vera* treated control;Ctp: cartap treated; Mtn: malathion treated; Ctp + Mtn: cartap + malathion treated; AE + Ctp: cartap with pretreatment of *A. vera*; AE + Mtn: malathion with pretreatment of *A. vera*; AE + Ctp + Mtn: cartap + malathion with pretreatment of *A. vera;* MDA: malondialdehyde; GSH: glutathione; ns: nonsignificant; * *p* < 0.05; ** *p* < 0.005; *** *p* < 0005.

**Table 4:** Effect of cartap and malathion as well as *A. vera* extract on the levels of change in normal architectural of rat liver in the different treatment groups.

P	C	AE + C	Ctp	Mtn	Ctp + Mtn	AE + Ctp	AE + Mtn	AE + Ctp + Mtn
**DH**	-	-	++	+++	+++	-	+	+
**CNH**	-	-	+++	+++	+++	-	+	+
**H**	-	-	+++	++	++	+	+	+
**CCv**	-	-	+++	++	++	-	+	-
**VD**	-	-	++	+	+	-	+	-
**DHt**	-	-	+++	+++	+++	+	+	+

Abbreviations: P: parameters; C: control; AE + C: *A. vera* treated control; Ctp: cartap challenged; Mtn: malathion challenged; Ctp + Mtn: cartap + malathion challenged; AE + Ctp: cartap with pretreatment of *A. vera*; AE + Mtn: malathion with pretreatment of *A. vera*; AE + Ctp + Mtn: cartap + malathion with pretreatment of *A. vera*; DH: degenerations of hepatocytes; CNH: coagulative necrosis in hepatocytes; H: degree of hemorrhage; CCv: congestion in central vein; VD: vacuolar degeneration; DHt: degeneration in hepatic triad. Each liver slide was examined, and the severity of the changes were scored using the scale of absent (-); mild (+); moderate (++) and severe (+++).
